# The courage to change science

**DOI:** 10.15252/embr.202050124

**Published:** 2020-02-20

**Authors:** Tiago Fleming Outeiro

**Affiliations:** ^1^ Department of Experimental Neurodegeneration Center for Biostructural Imaging of Neurodegeneration University Medical Center Goettingen Goettingen Germany; ^2^ Max Planck Institute for Experimental Medicine Göttingen Germany; ^3^ Translational and Clinical Research Institute Faculty of Medical Sciences Newcastle University Newcastle upon Tyne UK

**Keywords:** S&S: Ethics, S&S: Economics & Business

## Abstract

Bureaucracy, performance assessment and other pressures increasingly encroach on scientists’ ability to do science. The research community as a whole needs to address these perils.

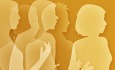

Much has been said and written about impact factors, h‐factors, citation scores, altimetrics and all the other measures to evaluate scientists and their performance. Much has also been said and written about the pressure to publish (or perish), career uncertainties, a dangerously skewed work–life balance, precarious funding and ever‐expanding bureaucracy in academic research. And yet, things have gotten worse. So why is it that we scientists continue to be evaluated in ways that are not scientific at all, and have to deal with increasing pressure and an ever‐expanding bureaucracy that wastes valuable time and energy better spent on research?

I have been a scientist for more than 20 years, and I still maintain the same excitement about science I had when I started. Most scientists are compelled to work hard to seek answers to baffling questions and problems, and often compromise their personal lives, their own health and their family life in their pursuit of truth. This is certainly not for the money, since there are many other jobs that pay much more for the same work and offer much better career options and job security.

I trust that most of my colleagues are honest, ambitious, dedicated and hard‐working researchers; yet, certain funding agencies and university administrations see us as suspicious characters who have to be vetted and controlled before they can trust us with even the merest amount of research funds. Unfortunately, there have been reasons for their distrust, but those cases of fraud and misconduct were by far the exception. It is important to understand why those happened though so we can change the system in order to avoid them in the future.

To start with, scientists are often overloaded with unrelated tasks and bureaucracy that go much beyond the research that they nonetheless have to do. For example, the number of forms to justify many of our research activities—even simple things such as sending material to colleagues or ordering life specimen—is not only absurd but, at times, offensive. We have to fill out forms to justify going to conferences, to get reimbursed for money we have to spend in advance or to ask for vacation. We have to fill out complex forms for conducting animal research, for using radionucleotides or toxic chemicals, or for using human cell lines or tissues. I understand the need for accountability and for justifying how we use public funding. However, what I find puzzling and frustrating is why our own academic institutions allow bureaucrats to flood us with ever more forms that no one will ever read. Why don't they find simpler and more efficient ways for keeping track of things instead of coming up with new bureaucratic hurdles?

Another essential question that we, as a community, need to address is why we tolerate that we are evaluated in ways most would agree are not accurate and, many times, not even fair? Here, I explore some ideas I have gathered over the years, based on my own experiences in different countries and through conversations with many colleagues from around the world.

Throughout our scientific careers, we are under constant pressure and stress. Pressure and stress are a hallmark of many professions and, within reasonable levels, not necessarily a bad thing. Stress keeps us on our toes. Being evaluated for our performance is part of life and part of science. The problem is that scientists are under pressure that is not productive for research and that can even be destructive, especially for junior scientists starting their independent careers.

We all face the pressure to publish to demonstrate productivity in order to get funding that will, again, allow us to publish new findings. The problem is that publishing is not enough. We are expected to publish in journals ranked according to metrics that can be manipulated, and the actual content of the publication often seems to be secondary to the title on the cover of the journal. The San Francisco Declaration on Research Assessment (DORA), along with other initiatives, has proposed a series of recommendations that should change the way scientists evaluate and are evaluated, so hopefully things will change over time.

Science is expensive, and funding is extremely competitive. Some colleagues have even suggested that funding agencies should attribute money through a lottery system, rather than a lengthy and costly review process that takes up much of reviewers’ time and often lacks objectivity owing to the low funding rates by most agencies. The extreme competitiveness creates more pressure, which creates more problems. Some scientists do not share their results before publication out of fear that others will take their data and publish first, which would prevent them from obtaining funding for continuing their work. The pressure also causes some scientists to cut corners, to overinterpret their findings or even manipulate their data so as to get published—which we all agree is unacceptable. We need to be aware of this problem and the reasons for it so we can discuss the issues and the best ways to solve them.

Another common problem many of us have experienced is the cruelty of peer review. This is a necessary process, and we participate since peer review is, despite its shortcomings, the best way to evaluate the ideas and work of others. Journals and funding agencies use different approaches, but the vast majority rely on anonymous peer review: our work is evaluated by our peers, who know who we are, whereas we are not allowed to know who they are. Being reviewed is not a bad thing and reviewers’ criticism and comments often help to improve our work. The problem is when people let their fear over competition take over, and stop being objective and reasonable, and use inconsiderate language. This is often meant to “kill” a paper or a grant, just because. Although increasing, this is still the exception though; fortunately, many colleagues maintain the fairness and collegiality that peer review calls for. It is nonetheless a calamitous symptom of the increasing pressure on scientists to perform. We should therefore push for more transparency and openness in the peer review process, as some journals already do. This would not solve all issues, but serve as a reminder that inconsiderate language will be seen by the whole community, and it might help reviewers focus on the science, using a constructive tone that should be the aim of the peer review, rather than “peer destroy”.

So what can we do to address these issues? The list of possible remedies is long, and no one so far has a magic bullet. I do not claim that I have a solution either, notably since the policies of journals, funding agencies or hiring committees are beyond the influence of individual scientists. But as a community, we have the means to change things. I would therefore like to share a thought rather than some concrete advice that could become the basis for all the other changes that are necessary: let scientists be scientists again. We need ways to evaluate productivity without obsessing with impact factors or journal name. We need new and creative policies to nurture junior scientists so they can excel and follow their passion for science. We should not allow reviewers to distil their frustration during the review process of a grant or manuscript. We need to be more collegial again and share findings and materials without fear of getting scooped. We should no longer need to fantasize on the impact of our findings, like we are often asked to do by funding agencies that have little clue about how science really works. This is even more relevant when we consider the impact of biomedical research on the lives of patients and their families. Overall, we need to be more objective again and focus on science and research. I think the general exasperation has grown so much that we need to move from words to actions, to experiment with the system and change the way we do things, so that science can take centre stage again in science.

